# Editorial

**Published:** 1841-11-06

**Authors:** 


THE MEDICAL EXAMINER.

PHILADELPHIA, NOV. 6, 1841.

### ARRANGEMENTS FOR THE ENSUING YEAR.

On and after the first of Jauuary, 1842, the present series of the Medical Examiner will be discontinued. A new series of the Examiner will be published in a form considerably altered from that now in use. It will be conducted by Dr. Reynell Coates, who will be the acting editor. Drs. Biddle and Gerhard will take particular charge of different departments of the Examiner, and the assistance of able collaborators, whose names or initials will be appended to the articles written by them, is secured.

It is proposed to restrict the foreign and selected matter within less space than they now occupy in the Medical Examiner, to give condensed views of the progress of medicine and the collateral sciences, bibliographical notices of new works of interest, reports of hospital and other cases, and original articles of a condensed and instructive character. In other words, the publishers of the new series of the Examiner will desire to keep their readers informed of all important medical novelties which may appear from time to time.

The Journal will be published every Saturday, by Joseph C. Turnpenny, (corner of Spruce and Tenth streets,) for the proprietors. Each number will consist of a single sheet, without a cover, by which arrangement distant subscribers will be subjected to newspaper postage only.

The subscription will be reduced to three dollars per annum for a single copy, and two copies to the same address will be forwarded regularly on the reception of an inclosure of five dollars.

The plan of payment of subscriptions in advance will be regularly adhered to, hereafter, as the only one consistent with the success of the undertaking in its altered form. Gentlemen remitting five dollars in advance, will be entitled to a single copy of the Journal for two consecutive years.

The announcement of a projected change in the editorship of this journal having been given above, it will be proper that we should call the attention of our readers from time to time to the views designed to be supported and the changes of arrangement to be introduced in the forthcoming New Series. Some of these changes will be gradually adopted in the succeeding numbers of the Examiner for the present year—and, as performances are better than promises, this will give our present subscribers, and others at a distance, the most available opportunity of judging the advantages likely to result from the alteration of plan.

Reference to the pages of the last and present numbers will show that it is a part of our design to be select in the choice of foreign articles, and that, while endeavouring to touch upon all subjects fitted for reception in a weekly journal that are of real importance and interest, we shall be somewhat more liberal in the expenditure of time and thought in commenting upon the contents of these articles than most editors permit themselves to be. In other words, the Foreign Department will assume more nearly the characters of critical and analytical bibliography. That it should do so, is desirable for the protection of human life against the influence of the false lustre acquired by many beautiful carvers, who carry the love of operating beyond all reasonable grounds, ingenious practitioners who are constantly introducing novelties in practice which will not bear the test of an examination regulated upon established principles, and medical theorists who become devoted to the study of the pathology of some one system of organs, and endeavour to employ the language, or rather, *the mere provincial dialect* of those organs in the explanation of the philosophy of morbid actions in other systems and tissues—a habit that is destructive of all correct generalization, and lays the foundation of much of the uncertainty in medical reasoning which has ever been regarded as one of the chief opprobria of the profession.

It will be observed, also, that a new department has been added in the present number, to embrace the proceedings of societies directly or indirectly connected with the cultivation of medical science. We should be pleased to extend this labour to other cities, if suitable arrangements could be effected for the purpose.

MEDICAL SCHOOL OF PHILADELPHIA.

The collegiate medical schools of late years, have engrossed so completely the title of "Medical Schools" *par eminence*, that the custom of applying the term in its narrowest acceptation has extended itself habitually to the press. We wish it distinctly understood, that, when we speak of *a medical school*, wo do not refer to any particular college or university, but to *all* the institutions collectively which contribute, or should contribute to the perfection of medical instruction in any one city, or around any one centre. Nor do we mean to exclude the private preceptors and their labours, which often form no unimportant feature in the system. These comments are called for in consequence of the uncorrected error of the press, in our last number, by means of which the heading of our article on this subject is graced with a pleural termination. After the clinics, which, *if rightly regulated*, are the most important feature in all medical schools, perhaps the colleges aud universities take the highest rank; but with us, there are numerous private establishments and lectureships which, in common with these heavier corporations, take rank, and sometimes an important rank in the system of instruction. In the next, or its succeeding number, we will endeavour to take some notice of these adjuvants.

During the last week we enjoyed the satisfaction of listening to the introductory lectures of Dr. Samuel Jackson and Dr. T. D. Mütter. By a resolution of the class of the University of Pennsylvania, five of the introductory addresses delivered at that institution which present a novel character, will be shortly published. As the lecture of Dr. Jackson will be included in this number, we defer most critical remarks until the whole are laid before us. It will be a subject of regret if the classes of the two other colleges should fail to copy this praiseworthy example; for even the most industrious editor cannot make himself omnipresent.

Of Dr. Jackson's lecture we will only remark at present that it was delivered in his usually lively style, and was more free than usual from his prevailing faults—unnecessary technicality, and an involution of phraseology. It contained more good old Saxon English than any other address from the same source we remember to have heard. On some other occasion we will notice its more solid merits, which were not slender, reserving still the right of criticism.

Dr. Mütter's lecture derives additional interest from the fact of its being his first essay since the assumption of the professional gown. It was classical in diction, and was delivered in a remarkably eloquent manner. Dr. M. is evidently practised in the art of oratory, and both voice and gesticulation were well ordered and impresssive. He dwelt with great force upon the broad distinction between the mere man of the knife and the real surgeon, and repudiated the culpable haste with which operations have been recently performed by those who prefer notoriety and gold won from the vulgar and ignorant, to real reputation among the wise and good, and the self-respect which flows from the consciousness of having fulfilled the duties incumbent upon the practitioner of a self-sacrificing, benevolent, and truly liberal profession. There was no praise of the institution with which he was connected—no censure cast upon others—no catering to the perverted taste of youthful dissipation by ill timed jest, or humourous and racy illustration. The tone of the address was decidedly grave and moral, and if it had a decided fault, it consisted in dwelling somewhat more on subjects of a religious character than the occasion seemed to require.

---

### Animal Magnetism.

The town has been lately much agitated by the public exhibition of experiments connected with Mesmerism, and, as usual on such occasions, the gossiping world is chiefly divided into two general classes—those whose credulity induces them to believe every thing seemingly wonderful that they see; devouring, wholesale, both facts and doctrines, and those so constitutionally sceptical or suspicious that they deny, without any *reasonable* examination, both the doctrines and the facts.

Between these contending factions, there are a set of observers—some of them *haud ignotus*—who gaze and wonder, wisely shaking their heads, and privately avowing that large pecuniary considerations would not induce them to confess how much they believe!

Among the public displays of the effects of certain manipulations exhibited in the recent demonstrations at the Masonic Hall, the majority of apparent phenomena might very well result from an ingenious system of collusion between the operator and the patient. That such collusion has existed in many of the displays of a similar character that have been made from time to time, both in this country and in Europe, there can be no doubt. The fact has been repeatedly proved. That both a real and a pretended effect has been occasionally combined in these public exhibitions—of which we seriously disapprove—there is little reason to doubt. All that relates to the apparent community of senses between the subject and the magnetiser, (so called unwisely, for there is no more proof that the phenomena are magnetic than there is that the maelstroom is the result of the gambols of a great submarine monster,) *may be effected* by a secret system of signs. The same is true of all that relates to the motions of the sleeper under the seeming influence of the will of another—such as the standing up, the clasping of hands, the raising of the arm, the working to time, &c.—experiments familiar to all—*these may be accomplished* by similar means. We should find it no difficult task to contrive such a system ourselves; and it would not require half the genius of Champollion in translating the Egyptian Hieroglyphics, Mr. Duponceau in discovering the history of Chinese Characters, *or Mr. Poe in decyphering secret writing*. Many of the startling descriptions *attributed to clairvoyance may be also* examples of the art of *guessing*. The whole subject furnishes a beautiful field for charlatanry, and we doubt not that many of our ingenious countrymen have found it a profitable business to gull our exceedingly gullable public in this manner. But that the operators at present engaged in the proceedings at the Hall are guilty of this species of collusion, we do not believe for many reasons, among which one of the most valid is as follows:

When men design to deceive by any plan, they generally render that plan as effective and perfect as possible. If the plan be regulated by signs, those signs are either perceived or they are not perceived. If the former, the action indicated is performed at once and perfectly; as in the case of the *learned pigs* and *puppies of genius* who are educated to spell words and trace out countries on a map. If, on the contrary, the signs be unheard or misunderstood, either no act is performed or a regular *faux pas* is committed. But in the experiments at the Hall, the attempt to produce certain motions at the will of the magnetiser very often fails, to the great dissatisfaction of the audience, and at the imminent risk of disappointing the only possible calculation of a deceptive operator—*the accumulation of pence.* Now, these failures are evidently involuntary, because they are contrary to the interest of the parties. Ye twe have observed that, in every instance, when we have been present, there has been an obvious *attempt to perform the required motion*, and never have we observed either total inactivity or the evidence of a design to effect a feat of a different character from that desired by the magnetiser. The very nature of these failures, then, and more especially their frequency, furnish strong evidence of the good faith of the exhibitions.

It is not our intention at present to dilate very widely upon the history of the phenomena displayed in the singular aberration of the nervous functions common to hysteria, catalepsy, somnambulism, pseudo-religious excitement, and *pow-wow-ing*. These phenomena have been familiar to philosophers from the days when the council of Amphictions first played upon the credulity of Greece by means of the ravings of hysterical girls of the Delphic cave, to those when good old Cotton Mather uttered his anathemas against witchcraft, and thence to our own times, and the period of our own observations. All that we contend for is the reality and importance of these effects, which have repeatedly determined health and disease, life and death. Call them imaginative or not, they are sufficiently important to be worthy of calm, philosophical investigation; and that investigation promises to throw much light on the history of that occult class of vital operations which results from the nervous function.

The medical man who now disbelieves the cataleptic and spasmodic effects of certain grimaces and gesticulations, acting on certain susceptible persons, betrays a culpable ignorance of his profession; and he who, *believing*, refuses to avow that belief for fear of public opinion, betrays a ridiculous degree of moral cowardice.

Dr. Wood, in his introductory of this year, very properly considers these aberrations as legitimate subjects for philosophical research, and acknowledges that the physical influence of the nervous system of one individual over another, is not absolutely beyond the bounds of physical possibility; but he considers this influence as effective only in weak minded subjects, and particularly women. We will quote one observation, in disproof of this, from among many at command.

Ultra immaterialist as we are, we entertain no idea that these artificial productions of nervous diseases are due to a metaphysical cause. Another observation will be given, showing most conclusively that the body, and not the mind of the operator, is the direct agent in producing the effect, and that the body and not the mind of the magnitised receives the immediate impression.

*Obs.* 1. A gentleman of deservedly high standing in the literary world, underwent a private application of the unintelligible process of the magnetiser, in our presence, a few days ago. He is a man of great firmness and decision of character, and altogether above the suspicion of lending himself to charlatanry. It was found impossible to effect his mind, but not so his person. In a very few minutes his eyes involuntarily closed, and the expression of his face was totally changed. After the vain endeavour to produce a farther impression, the operation ceased. He was asked *by a bystander* whether he heard what was going on around him. He made violent efforts to reply, throwing his face into strange contortions, but was utterly unable to open his mouth. He attempted to join in the laugh occasioned by his ludicrous condition, but the muscles refused their office and the contortions of the countenance were redoubled. He was requested to open his eyes, but all his efforts were useless. Making signs for writing materials, on receiving them he proceeded to describe his sensations, and had completed two well written lines, when he recovered almost instantly. He then stated that this was the third time that he had been "magnetised," at the distance of several years, and by different individuals, but always with precisely the same results. His sense of sight appeared to him to be totally lost, while that of hearing was redoubled in intensity, and he was utterly incapable of speaking; but his mind was totally unaffected.

*Obs.* 2. The arm of a young lady was separately acted upon in the extended position, and the extensors were rendered very rigid. The arm was carried horizontally outwards. The influence did not extend to the deltoid, which soon became paralysed so as to require that the elbow should be supported. Sensation in the part was totally lost in the forearm, so that a deep scratch, an inch in length, made with a pen-knife, and which bled very freely, (a most inexcusable experiment) awakened no emotion. We placed our left hand upon the bend of the elbow, and, with the right, steadily exerted a force of about sixty pounds for nearly two minutes, in the attempt to bend the elbow, but without producing the slightest impression. The moment we relaxed the force, all the muscles of the forearm were thrown into one of the most violent tetanic spasms that we ever witnessed. The hand became perfectly livid from venous engorgement, and the fingers,—each joint half flexed,—were rigid as iron. The spasm very soon extended to the biceps and deltoid; which were still sensible; and when the magnetiser was suddenly called to the young lady's relief, she was on the point of screaming with pain. There appeared to be real danger of rupture of the tendons. Now the total absence of unusual mental phenomena in this case—for, until the occurrence of the last spasm, she was laughing and talking with a friend in a lively manner—distinctly proves that the phenomena are not, as certain miscalled theorists pretend, the result of the action of mind on mind; but they also show the folly of those who fly from the investigation of important facts because of the mass of unmeaning nonsense with which those facts are loaded by a few wild dreamers.

So far as the writings of the various observers are concerned, not one ray of light has yet been thrown upon the facts of the question. They stand detached and isolated from our present knowledge; but we believe that many even of the most remarkable of them may be reduced within physiological rule, and, on some future occasion, we propose to enlarge upon the subject. R. C.

---

The article on Tenotomy is necessarily deferred until the appearance of the next number.

